# *Serratia marcescens* corneal abscess with pink biofilm clue

**DOI:** 10.1016/j.ajoc.2026.102646

**Published:** 2026-08-26

**Authors:** Victor Codina Alonso, David Lopez Oliver, Horace Massa

**Affiliations:** a Department of Ophthalmology, Geneva University Hospitals, Geneva, Switzerland; b Faculty of Medicine of the University of Geneva, University of Geneva (UNIGE), Geneva, Switzerland

**Keywords:** Contact lens, Corneal abscess, Corneal infection, Prodigiosin, *Serratia marcescens*

## 1 Case report

**Fig. 1:**
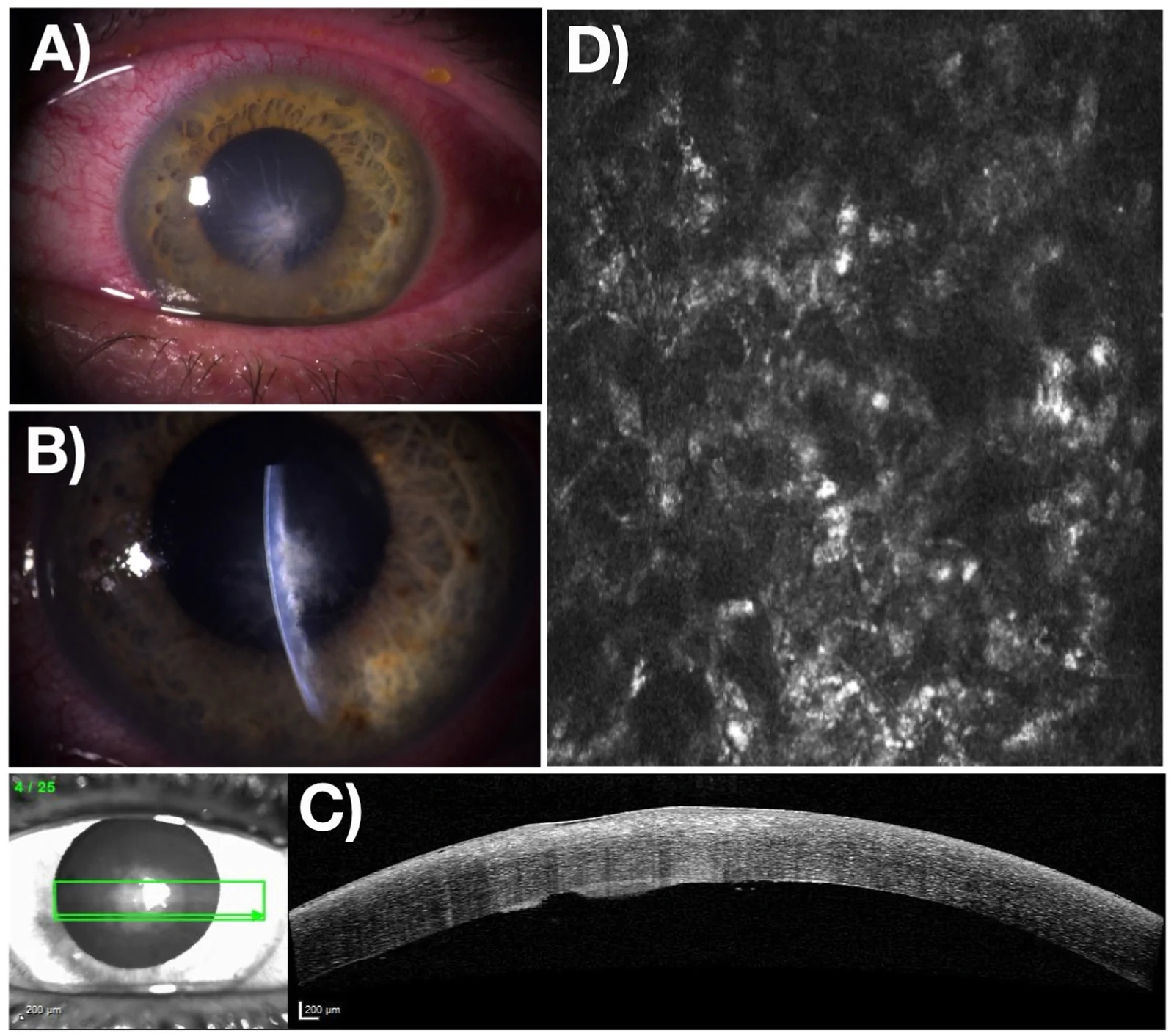
please replace the first line with “Initial presentation of a 38-year-old woman soft contact lens user who presented with a painful red left eye, purulent discharge and blurred vision who was later diagnosed with *Serratia marcescens* keratitis.”. (For interpretation of the references to colour in this figure legend, the reader is referred to the Web version of this article.)

**Fig. 2:**
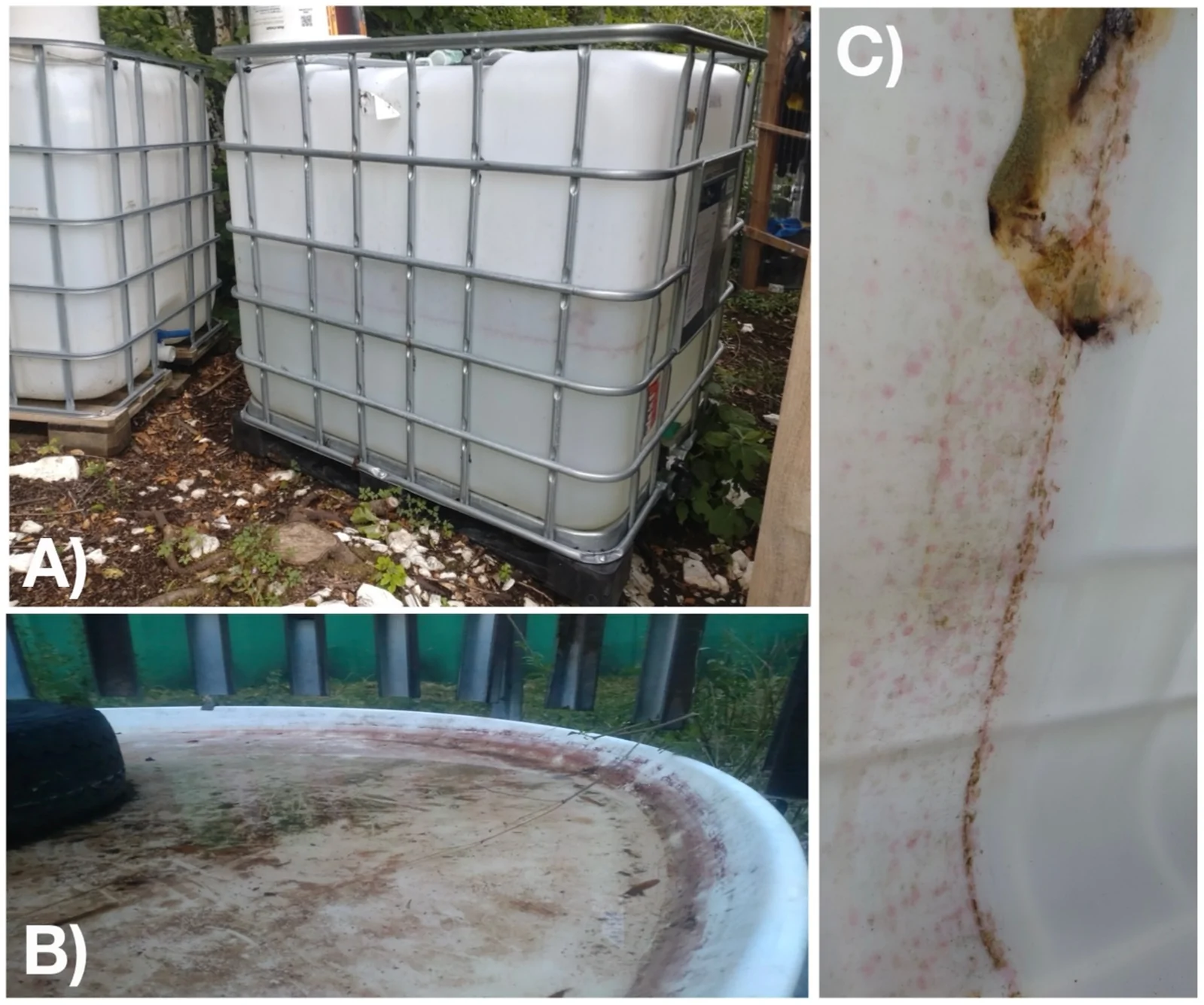
(A and B) change “photograph” to “photographs”.

**Fig. 3:**
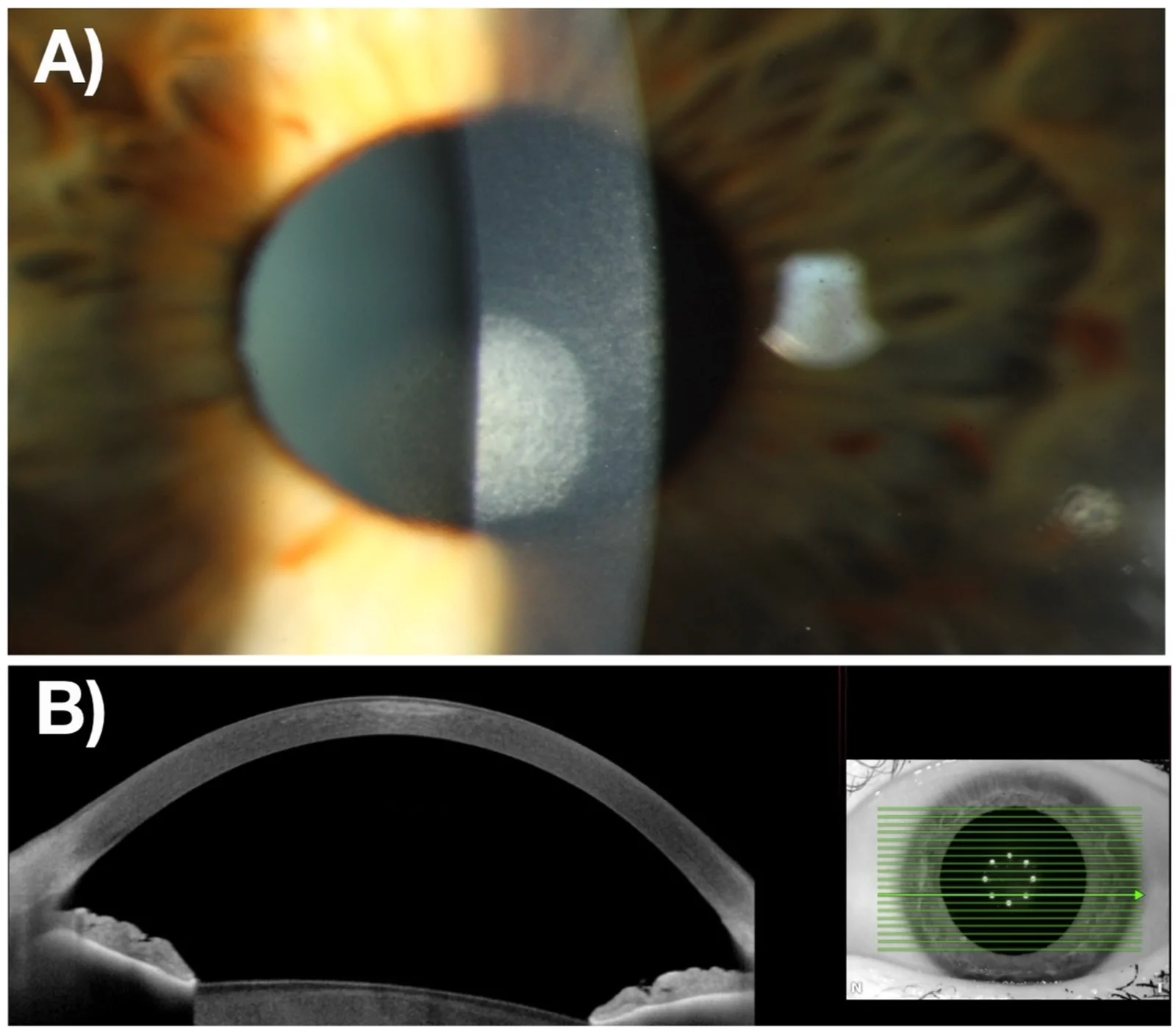
please replace the first line with “Anterior segment images of the left eye after 6 weeks of antimicrobial therapy for *Serratia marcescens* keratitis in a 38-year-old woman soft contact lens user”.

A 38-year-old woman wearing soft contact lenses presented to the ophthalmology emergency department with a two-day history of a painful red left eye, purulent discharge and blurred vision after showering with rainwater stored in a farm water tank. On initial examination, best-corrected visual acuity was 20/20 in the right eye and 20/200 in the left eye. Slit-lamp biomicroscopy of the left eye revealed pronounced severe conjunctival and limbal injection, a fibrinous reaction with +2 cells within the anterior chamber, and a well-circumscribed, centrally located corneal stromal infiltrate measuring approximately 2 mm × 2 mm, accompanied by an overlying epithelial defect and positive fluorescein staining (Fig. 1A and B). Anterior segment optical coherence tomography (AS-OCT) confirmed stromal involvement with an associated endothelial plaque (Fig. 1C). Confocal microscopy demonstrated intense stromal inflammation with hyperreflective inflammatory cells, without hyphae or cysts suggestive of *Acanthamoeba* (Fig. 1D). Detailed environmental anamnesis revealed exposure to a farm water tank containing visible pink biofilm (Fig. 2). Corneal scraping was performed, and the patient was treated with hourly topical moxifloxacin (0.5%) and topical tobramycin (0.3%). Cultures identified *Serratia marcescens,* and antibiotic sensitivity testing confirmed susceptibility to fluoroquinolones and aminoglycosides. Treatment frequency was later tapered according to clinical response. Complete resolution was achieved after 6 weeks of treatment, with final best-corrected visual acuity of 20/25 in the left eye. Follow-up slit-lamp examination showed resolution of the stromal infiltrate with minimal residual anterior stromal scarring, absence of further fluorescein staining, resolved conjunctival injection, and no cells in the anterior chamber (Fig. 3A). AS-OCT confirmed normalization of stromal architecture and disappearance of the endothelial plaque indicating a favorable clinical and functional outcome (Fig. 3B).

## 2 Discussion

*Serratia marcescens* is an opportunistic Gram-negative pathogen known to cause contact lens-associated keratitis and corneal ulcers, often requiring culture-directed therapy.^1^, ^2^, ^3^ Previous reports have highlighted its association with improper lens hygiene and water exposure, as well as variable visual outcomes depending on early diagnosis and treatment.^1^^,^^3^ However, identification of a visible environmental source containing red-pigmented biofilm: *prodigiosin*, is rarely documented in clinical reports. To our knowledge, this is one of the few reported cases in which a visible environmental prodigiosin-associated biofilm provided an indirect diagnostic clue linking the environmental source to *Serratia marcescens* infection before microbiological confirmation. Neither the corneal lesion nor the ocular discharge in this case exhibited any such pigmentation. Otherwise, in this case the presence of pink biofilm on the contaminated water tank provided an immediate diagnostic clue, linking environmental exposure to the causative organism. Recognition of *prodigiosin-*producing biofilm can assist clinicians in narrowing the differential diagnosis in atypical corneal abscess presentations. In addition to antimicrobial therapy, the patient was advised to discontinue contact lens wear for 4 months, avoid any exposure to non-sterile water, and ensure proper hand hygiene before lens handling. Cleaning and disinfection of the farm water tank were recommended to prevent further contamination and recurrence. This finding reinforces the importance of a thorough environmental history, particularly in rural settings where non-sterile water may be used during hygiene practices.

## 3 Conclusion

Environmental anamnesis played a key role in identifying the infectious source in this case. The visible pink biofilm acted as a clinically relevant clue for *Serratia marcescens* infection, facilitating prompt targeted management. Contact lens users should be advised to avoid exposure to non-sterile water, especially in rural environments.

## CRediT authorship contribution statement

**Victor Codina Alonso:** Writing – original draft, Conceptualization. **David Lopez Oliver:** Supervision, Conceptualization. **Horace Massa:** Validation.

## Patient consent

Consent to publish this case report has been obtained from the patient(s) in writing.

## Authorship

All authors attest that they meet the current ICMJE criteria for Authorship.

## Funding

No funding or grant support.

## Declaration of competing interest

The authors declare that they have no known competing financial interests or personal relationships that could have appeared to influence the work reported in this paper.
